# Dexmedetomidine combined with local anesthetics in thoracic paravertebral block: A systematic review and meta-analysis of randomized controlled trials

**DOI:** 10.1097/MD.0000000000013164

**Published:** 2018-11-16

**Authors:** Kai Wang, Li-jun Wang, Tong-jiu Yang, Qing-xiang Mao, Zhen Wang, Li-yong Chen

**Affiliations:** a Department of Anesthesiology, Daping Hospital, Institute of Surgery Research, the Army Medical University, Chongqing; b Department of Anesthesiology, 535 Hospital of PLA, Huaihua, China.

**Keywords:** anesthesia adjuvant, dexmedetomidine, paravertebral block

## Abstract

**Background:**

Dexmedetomidine (DEX) improves postoperative pain scores and prolongs the duration of blockage when combined with local anesthetics (LAs) for neuraxial and brachial plexus block; however, there is little information about the effectiveness of DEX as an adjuvant to LAs in paravertebral block (PVB). Therefore, a systematic review and meta-analysis were performed to evaluate the safety and efficacy of DEX combined with LAs in PVB.

**Method:**

An electronic database search from inception date to February 2018 was performed. Randomized controlled trials (RCTs) comparing DEX as an adjuvant to LAs with LAs alone for PVB in adult patients were included. Postoperative pain scores, duration of analgesia, cumulative perioperative analgesic consumption, and adverse events were analyzed.

**Result:**

We identified 7 trials enrolling 350 patients and found that DEX reduced pain scores at rest by standardized mean differences (SMD) −0.86 cm (95% confidence interval [CI] [−1.55, −0.17], *P* = .01) and SMD −0.93 cm (95% CI [−1.41, −0.26], *P* =.008) at postoperative 12 hours and 24 hours, respectively. DEX reduced pain scores while dynamic by SMD −1.63 cm (95% CI [−2.92, −0.34], *P* =.01) and SMD −1.78 cm (95% CI [−2.66, −0.90], *P* =.007) for postoperative 12 hours and 24 hours, respectively. DEX extended the duration of analgesia by weighted mean differences (WMD) 201.53 minutes (95% CI [33.45, 369.61], *P* =.02); and reduced cumulative postoperative analgesic consumption by WMD −7.71 mg (95% CI [−10.64, −4.78], *P* <.001) and WMD −45.64 mg (95% CI [−69.76, −21.53], *P* < .001) for 24 hours morphine and 48 hours tramadol subgroups, respectively. DEX also increased the odds of hypotension by odds ratio (OR) 4.40 (95% CI [1.37, 14.17], *P* = .01); however, there was no statistically significant difference for intraoperative fentanyl consumption and the incidence of the bradycardia.

**Conclusions:**

DEX combined with LAs in PVB significantly improved postoperative pain scores, prolonged the duration of analgesia, reduced postoperative analgesic consumption, and increased the odds of hypotension. However, we cannot neglect the heterogeneity of the included RCTs. More large-scale prospective studies are needed to further clarify the above conclusions.

**Systematic review registration:**

PROSPERO registration number CRD42018090251.

## 1 Introduction

The increased popularity of the paravertebral block (PVB) can be attributed to its relative safety and efficacy. PVB has been studied as a potential replacement to epidural block analgesia, because it provides pain relief comparable with traditional epidural analgesia, and has reduced side effects.^[1]^ The application of various technical refinements and the enhanced efficacy and safety of the PVB make it suitable as the new standard for perioperative analgesia after the appropriate surgical trunk procedures.^[2]^ Increasing numbers of unilateral surgeries have used paravertebral blockade for perioperative analgesia, such as breast, chest wall, thoracotomy, and renal surgeries.^[3]^ However, the duration of current LAs is limited by analgesic advantages, particularly during postoperative analgesia. While a catheter can be placed in the paravertebral space for continuous postoperative pain control, this placement requires additional time and costs and increases the risk of infection and neurological complications. Therefore, anesthetists have sought strategies that prolong nerve blocks beyond the duration of current available LAs.^[4]^ Perineural adjuncts are a technically simple strategy that can be used for this purpose.^[5]^ For example, dexamethasone,^[6]^ fentanyl,^[7]^ and morphine^[8]^ have been demonstrated to extend the duration of PVB analgesia with varying efficacy.

Dexmedetomidine (DEX) is a highly selective alpha-2 adrenergic receptor agonist.^[9]^ The US Food and Drug Administration (FDA) has approved DEX delivery only via the intravenous route; however, anesthetists have employed DEX extensively for off-label indications. Three recent meta-analyses have demonstrated that DEX can accelerate the onset and extend the duration of blockade when combined with LAs for brachial plexus blockade.^[10–12]^ A further meta-analysis has demonstrated that DEX is a favorable adjuvant to LAs with better and longer analgesia for neuraxial blockade.^[13]^ The efficacy and safety of DEX combined with LAs is a hot research topic. However, there is little information about the effectiveness of DEX combined with LAs in PVB. Consequently, we have performed a systematic review and meta-analysis of the published studies to assess the safety and efficacy of DEX combined with LAs in PVB.

We performed a PICO (patient problem or population, intervention, comparison, and outcomes) analysis: PVB for unilateral surgeries in adult conditions (P) DEX as an adjuvant to local anesthetics (LAs) (I) compared with LA alone (C) resulting in ameliorated clinical outcomes (O).

## 2 Materials and methods

We registered the current meta-analysis at PROSPERO (CRD42018090251). The study was conducted in accordance with the references from Cochrane Collaboration^[14]^ and the guidelines from the Quality of Reporting of Meta-analyses (QUORUM).^[15]^ Both patient consent and ethical approval were not required because the meta-analysis was built on previously published literature.

### 2.1 Literature search

Two reviewers (WK and WLJ) independently sought and retrieved relevant studies from electronic databases, including PUBMED, MEDLINE, EMBASE, Web of Science, Cochrane Central Register of Controlled Trials and Cochrane Library. Controlled vocabulary terms, text words, and medical subject headings (MeSH) associated with DEX, Medetomidine, and Precedex were sought. We combined these results with search terms associated with PVB using the Boolean operator “AND”. Retrieval time was from the inception of the databases to 1 February 2018. We also considered the alternative spellings for keywords and searched for grey literature from other Internet resources.

### 2.2 Eligibility criteria

Inclusion criteria were as follows:

(1) Randomized controlled trials (RCTs);
(2) Comparison between LAs with DEX and LAs alone in any level of PVB (single shot or continuous catheter) for ipsilateral surgeries, including breast surgery, renal surgery, thoracotomy, laparoscopic and chest wall surgery;
(3) Adult patients;
(4) English language.

Exclusion criteria were as follows:

(1) non-RCTs;
(2) DEX administered intravenously;
(3) Comparison between LAs with DEX and LAs with other drugs;^[7,8]^
(4) Unpublished or in progress;
(5) Conference abstract.

### 2.3 Trial selection and quality appraisal

Two reviewers (WK and WLJ) independently applied inclusion criteria from a review of the titles, abstracts, and keywords. Inconsistencies were settled by discussion or through consultation with the third reviewer (YTJ) until a consensus was reached. References were then searched by hand by the third reviewer (YTJ).

The reviewers (WK and WLJ) independently evaluated the methodological quality of the included RCTs according to the guidelines in the Cochrane Reviewer's Handbook.^[16]^ Studies were assessed for random sequence generation, allocation concealment, blinding of participants and personnel, blinding of outcome assessors, incomplete outcome data, selective reporting, and any other potential source of bias. The results of every trial were used following consensus between the 2 reviewers. Inconsistencies were settled by discussion or through consultation with the third reviewer (YTJ) until a consensus was reached.

### 2.4 Data extraction and outcome assessment

The reviewers (WK and WLJ) independently extracted relevant data using a standardized data table. The extracted information included main author, publication year, groups, sample size, nature of primary outcome, nerve localization techniques, surgical location, dose of DEX (shown as dosages per average body weight), type and dose of LA, outcome (analgesic effects and DEX related side effects) definition, outcome units, and outcome data.

We used data that were presented in tables as the first provenience for extraction; when information was not reported in tables, we contacted original author for additional data. Considering the limited number of RCTs, trials reporting range or interquartile range (IQR) were included using an estimate of the standard deviation (SD) from the formulae: SD = Range/4 and SD = IQR/1.35, respectively, as described by the Cochrane Handbook.^[16]^ Data reported as 95% confidence intervals (CIs) were also used to estimate the range, which was then converted to SD. If the mean was not provided, the median was used to evaluate the quantitative value.^[17]^ When SD values were not reported for an outcome (e.g., postoperative pain), these values were imputed.^[18]^ When the data that were required were present in figures and the original data was not obtained from the authors, we extracted data from the published figures using Image J software (Image J software, National Institutes of Health, USA, http://imagej.nih.gov). In addition, we converted the dichotomous data with respect to the adverse effects to incidence (n/N) during the perioperative period.

We designated postoperative pain severity using the visual analogue scale (VAS: 0 = no pain, 10 = worst pain imaginable) during rest and dynamic at postoperative 12 hours and 24 hours, as the primary outcome. Secondary outcomes included the analgesic outcomes, duration of postoperative analgesia, cumulative postoperative analgesic consumption, intraoperative fentanyl consumption, patient satisfaction with postoperative pain relief, DEX related adverse effects^[19]^ (bradycardia, hypotension, excessive sedation, hypoxemia), and postoperative nausea and vomiting (PONV).

### 2.5 Predefined sources of heterogeneity

Considering the possible causes of heterogeneity in the final results, we preidentified the clinical features of each trial and known confounders that may result in variations in our primary outcome results. The variables of interest included:

(1) surgical location;
(2) time of surgery;
(3) LA type and dose;
(4) DEX dose;
(5) block localization technique; and
(6) PVB performed before induced anesthesia or at the end of the surgery.

### 2.6 Statistical analysis

One reviewer (WK) input the data and another (WLJ) checked its accuracy. Meta-analysis was implemented using Review Manager (RevMan for Windows, version 5.3, Cochrane Collaboration, Oxford, UK) to pool the data where possible. The summary measure was the standardized mean difference (SMD) for the postoperative pain score and mean difference (MD) for postoperative analgesic consumption, intraoperative fentanyl consumption, and duration of postoperative analgesia. The summary measure was the odds ratio (OR) for PONV and DEX related adverse effects. Subgroup analysis by postoperative rescue analgesia type (morphine, tramadol, and ropivacaine) and predefined sources of heterogeneity were performed.

Statistical significance was defined as when *P* < .05 and 95% CI ≠ 0 for SMD and MD, or 1 for odds ratio (OR). The heterogeneity of the pooled results was assessed using the I^2^ statistic.^[20]^ We explored the sources of heterogeneity by examining the association with predefined confounders if the heterogeneity was significant (I^2^ >50%).

## 3 Results

**Figure 1:**
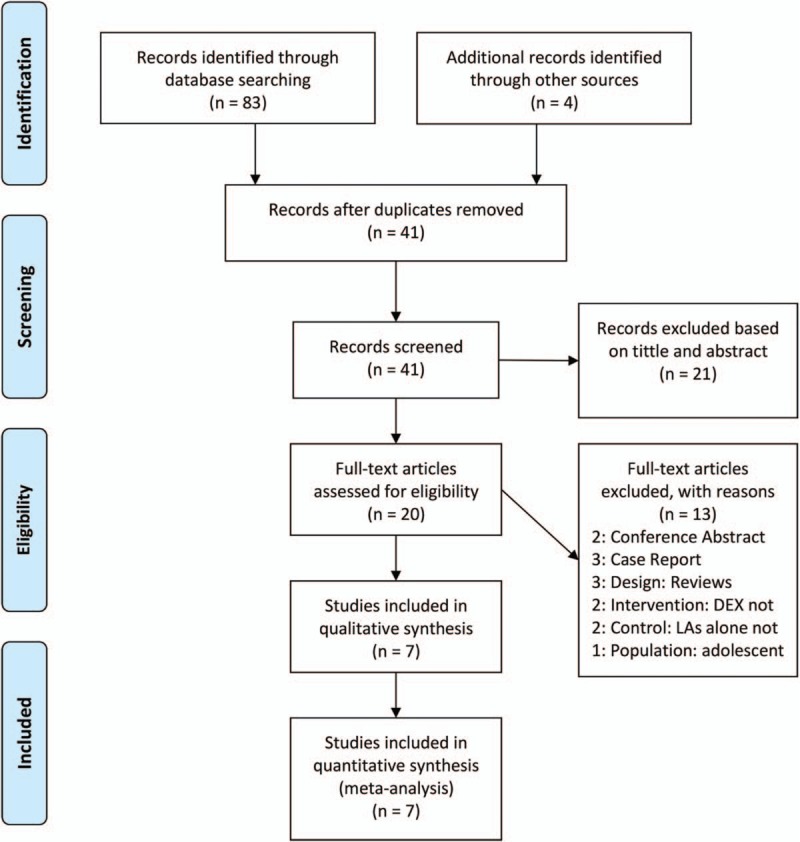
Flow diagram summarizing retrieved, included, and excluded trials.

We retrieved 87 potentially relevant records and removed 46 duplicates. After filtering the title and abstract, 21 studies were excluded. After reviewing the full text, 13 studies were excluded. Finally, 7 full-text RCTs^[21–27]^ were included. The flow diagram and main causes for exclusion records are represented in Figure 1. No additional study was found following a search by hand.

### 3.1 Trial characteristics

We extracted data from a total of 350 participants, including 175 in the DEX group and 175 in the Control group. Details of the 7 RCTs, country, surgery, groups, DEX dose, nerve block localization, sample size, PVB time, single injection or infusion, and primary outcomes assessed are represented in Table 1. Four trials were performed by single shot PVB^[22,23,26,27]^ and 3 trials inserted a continuous catheter inside the paravertebral space^[21,24,25]^ at the level of the surgical incision. Only 1 PVB was performed at the end of surgery,^[27]^ and the rest were implemented before general anesthesia. The nerve block localization technique used was anatomical (landmark) in 3 trials,^[21–23]^ ultrasound in 3 trials,^[24,25,27]^ and not defined in 1 trial.^[26]^ All trials used long acting LAs (ropivacaine or bupivacaine). DEX was used according to single doses per average body weight (1.0 μg/kg) and continuous doses (0.2 μg/kg/h).^[24,25]^ The control group in 2 trials were not LAs alone, but fentanyl^[7]^ and morphine,^[8]^ so the results were excluded from our analysis. All trials reported analgesic outcomes and dexmedetomidine-related complications.

**Table 1:**
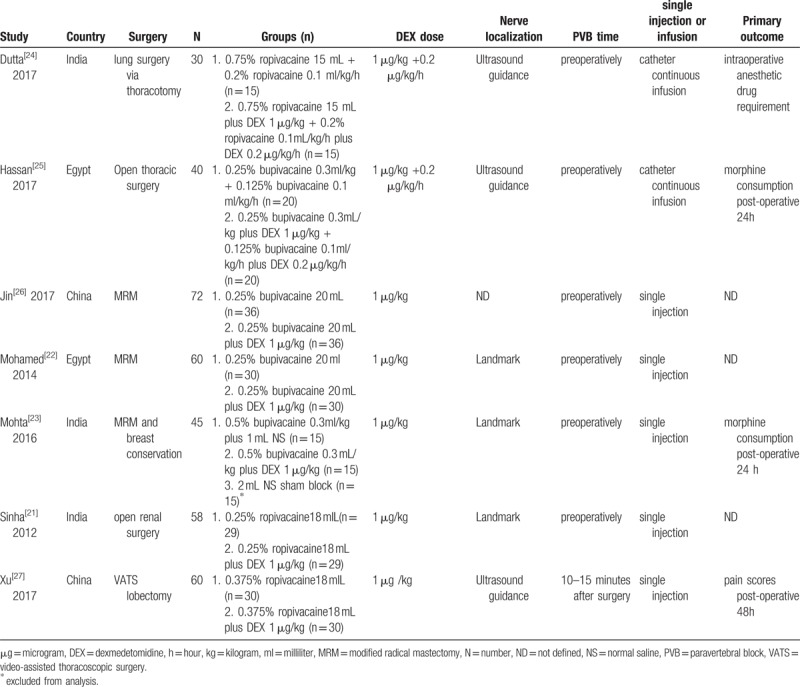
Trial characteristics and outcomes examined.

### 3.2 Risk of bias assessment

**Figure 2:**
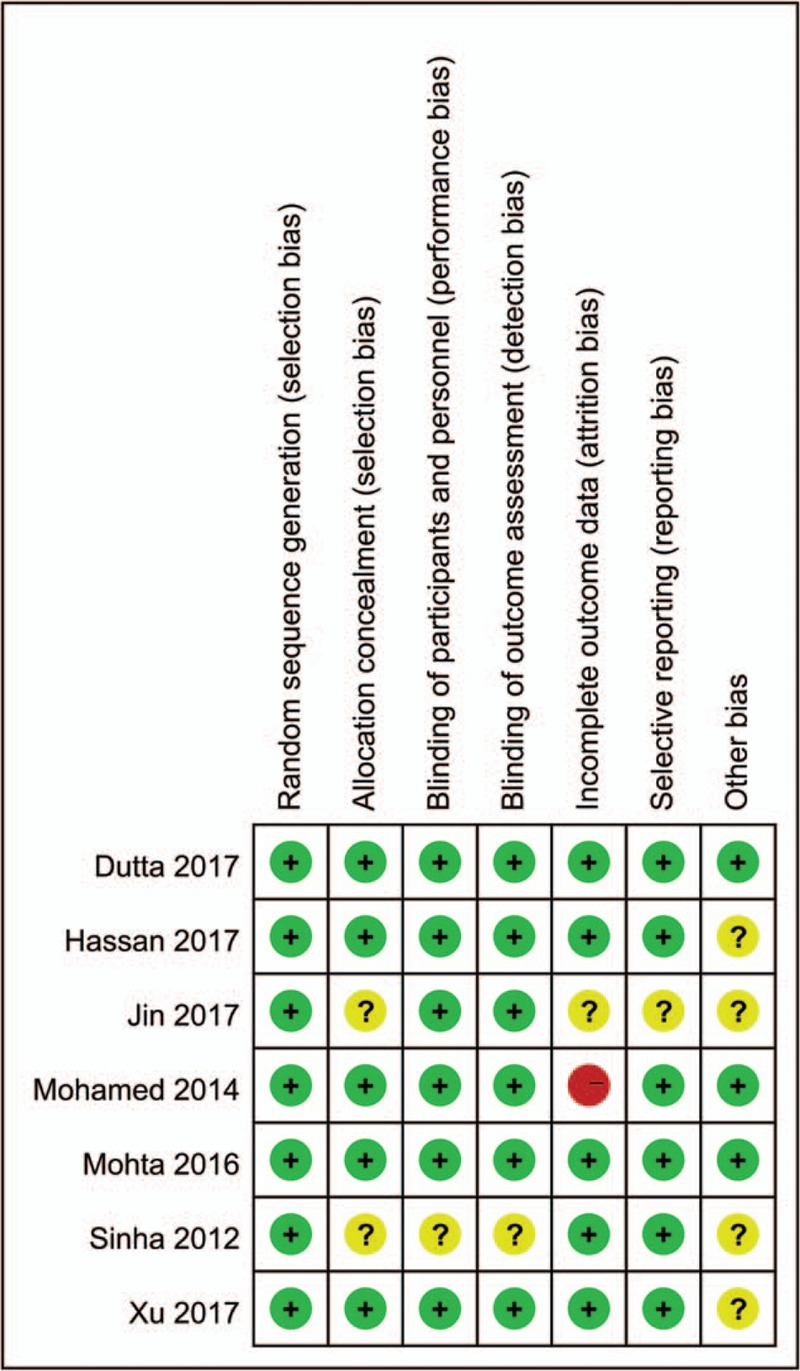
Risk of bias summary (red circle = high bias risk, green circle = low bias risk, yellow circle = unclear bias risk).

The reviewers’ consensus assessment results are represented in Figure 2. We considered the methodological quality for the majority of the 7 trials included to be acceptable and evaluated the overall risk of bias across the trials as moderate. All the trials distinctly represented the program of randomization. Most of RCTs had low risk for allocation concealment (for patients, researchers, and result assessment), and selection, performance, detection, attrition, and reporting biases. Moreover, few trials evaluated had an unclear risk of bias, because there were not sufficient details. Attrition bias was classified as high^[22]^ because there were not detail data about dexmedetomidine-related sedation scores.

### 3.3 Analgesic outcomes

#### 3.3.1 Postoperative pain scores

**Figure 3:**
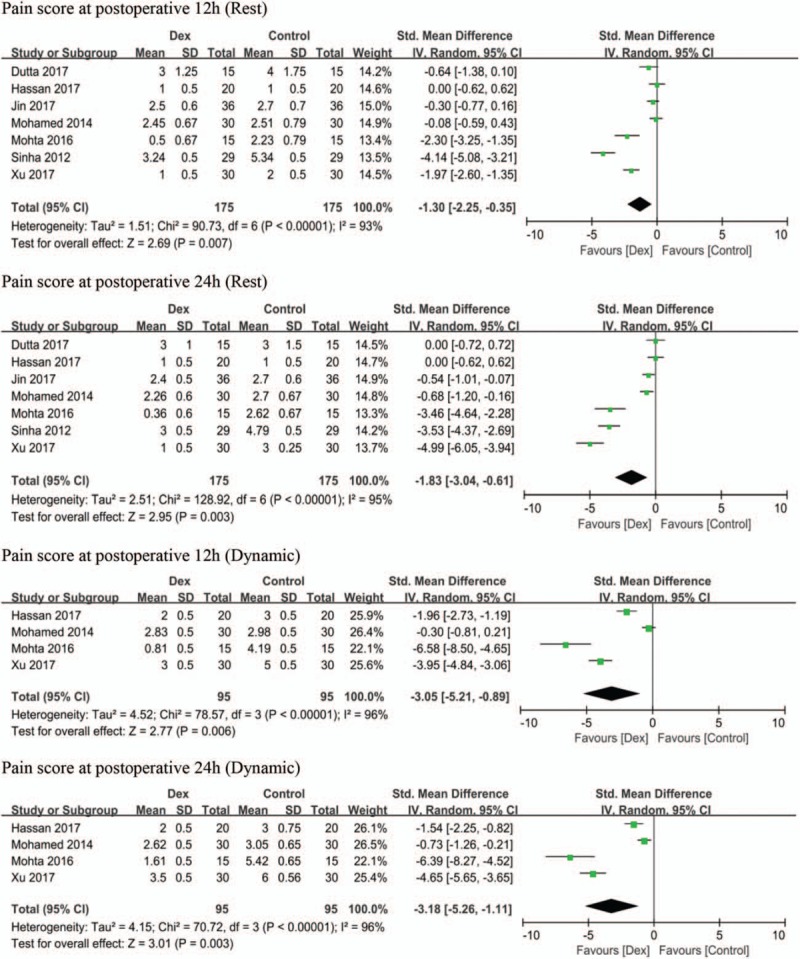
Forest plots comparing the effect of dexmedetomidine on postoperative pain scores at rest and dynamic after postoperative 12 hours and 24 h. DEX = dexmedetomidine, SD = standard deviation, CI = confidence interval.

All trials reported the primary outcome, postoperative pain score. The effect of DEX combined with LAs on postoperative pain scores at rest was reported in all trials, while dynamic pain scores were reported in 4 trials,^[22,23,25,27]^ with respect to pain assessment using the VAS^[21,22,24–26]^ and Numerical Rating Scale (NRS).^[23,27]^ Therefore, postoperative pain severity, reported as NRS score, was converted to VAS score.^[28]^ Pooled trials showed that DEX reduced the pain scores at rest by an SMD [95% CI] of −0.86 cm [−1.55, −0.17], (*P* =.01, I^2^ = 96%) and −0.93 cm [−1.41, −0.26], (*P* =.008, I^2^ = 97%) for postoperative 12 hours and 24 hours, respectively, and DEX reduced pain scores while dynamic by an MD [95% CI] of –1.63 cm [−2.92, −0.34], (*P* =.01, I^2^ = 99%) and −1.78 cm [−2.66, −0.90], (*P* =.007, I^2^ = 99%) for postoperative 12 hours and 24 hours, respectively. Figure 3 shows a forest plot for these data. Considering the significant heterogeneity (I^2^ ≥96%), further subgroup analysis of LA types, continuous or single shot PVB, nerve localization techniques, surgery types, and sensitivity analyses did not contribute to this heterogeneity (Table 2). Of note, the mean pain score from 2 studies^[22,23]^ were extracted as expected scores from published figures using Image J software because the raw data were not available. These data indicated that DEX as an LA adjuvant on PVB significantly improved postoperative pain scores while dynamic and rest, although inconsistency was high.

**Table 2:**
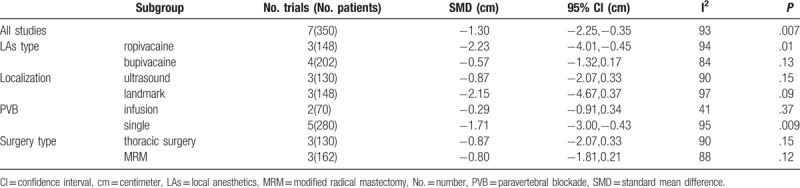
Subgroup analysis of dexmedetomidine on postoperative pain scores at rest (12hour).

#### 3.3.2 Intraoperative fentanyl consumption

**Figure 4:**
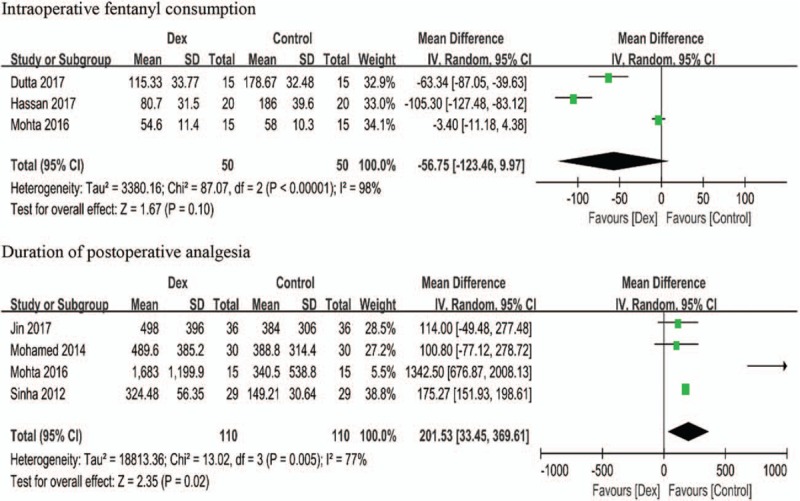
Forest plots intraoperative fentanyl consumption and duration of postoperative analgesia. DEX = dexmedetomidine, SD = standard deviation, CI = confidence interval.

Cumulative intraoperative fentanyl consumption was reported in 4 trials.^[23–25,27]^ PVB was implemented at the end of the surgery in only 1 trail;^[27]^ therefore, these data were not included. Pooled trials revealed no statistically significant difference in intraoperative fentanyl consumption, with a mean difference [95% CI] of −56.75 μg [−123.46, 9.97], (*P* =.10, I^2^ = 98%), as shown in Figure 4. We did not conduct further subgroup analysis because of the small number of trials.

#### 3.3.3 Duration of postoperative analgesia

The effect of combining DEX with LAs on the duration of analgesia was evaluated in 5 trials.^[21–23,26,27]^ The definition of duration of postoperative analgesia in these trials varied according to different hallmark events, including time to reach a VAS score >3,^[21]^ VAS ≥3,^[22]^ NRS >3,^[23]^ NRS ≥4^[27]^ at rest, and patient first requesting medicine for postoperative pain at surgical incision. In addition, duration of postoperative analgesia was not defined in 1 trial.^[26]^ Administration of 100 mg intravenous (IV) flurbiprofen every 12 hours for 3 days was used as the routine postoperative analgesic, and rescue analgesia was applied to 1 patient in each group, at postoperative 36 and 17 hours in the DEX and Control group, respectively;^[27]^ therefore, these data were not included. Pooled trials showed that combining DEX with LAs extended the duration of analgesia by an MD [95% CI] of 201.53 minutes [33.45, 369.61], (*P* =.02, I^2^ = 77%), as shown in Figure 4.

#### 3.3.4 Cumulative postoperative analgesic consumption

**Figure 5:**
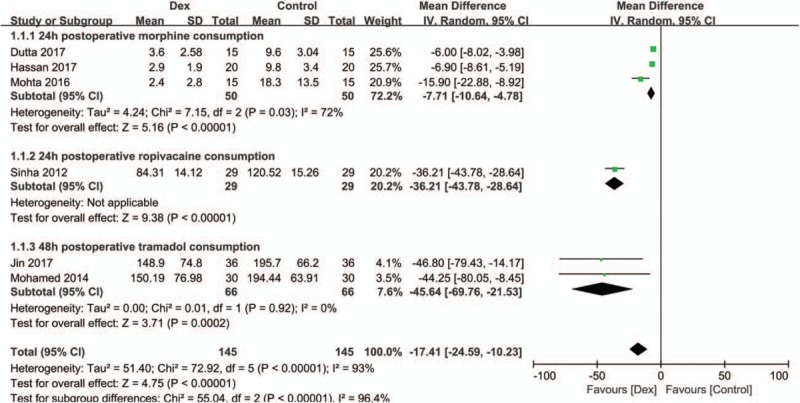
Forest plots cumulative postoperative analgesic consumption. DEX = dexmedetomidine, SD = standard deviation, CI = confidence interval.

Cumulative postoperative analgesic consumption was reported in all trials. Cumulative 24 hours postoperative morphine consumption was reported in 3 trials,^[23–25]^ total ropivacaine^[21]^ consumption was recorded in the first 24 hours, and morphine^[27]^ and tramadol^[22,26]^ requirements were recorded in the first 48 hours of the postoperative period. Administration of 100 mg flurbiprofen (IV) every 12 hours for 3 days was the routine postoperative analgesic, and rescue morphine was applied to 1 patient in each group;^[27]^ therefore, these data were excluded. These data revealed that combining DEX with LAs reduced cumulative postoperative analgesic consumption by an MD [95% CI] of −7.71 mg [−10.64, −4.78], (*P* <.001, I^2^ = 72%) and −45.64 mg [−69.76, −21.53], (*P* <.001, I^2^ = 0) for the 24 hours morphine and 48 hours tramadol subgroups, respectively. These data are shown in Figure 5.

#### 3.3.5 Patient satisfaction with pain management

Patient satisfaction with pain management was assessed in 3 trials,^[23,24,27]^ using the VAS scale (0–10, 0 being unsatisfied and 10 being fully satisfied);^[24]^ 3 point scale,^[23]^ and 5-point Likert scale.^[27]^ The patients’ satisfaction about postoperative pain management was significantly higher in the DEX group than in the control group in the 3 trials.

#### 3.3.6 Adverse effects

**Figure 6:**
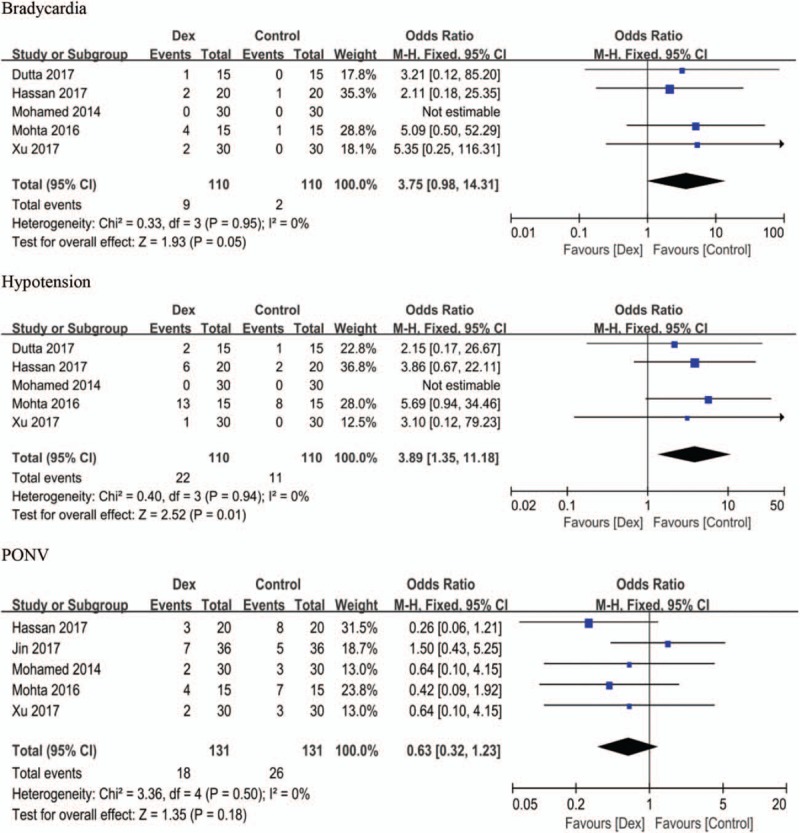
Forest plots adverse effects. DEX = dexmedetomidine, CI = confidence interval, PONV = postoperative nausea and vomiting.

The definitions of DEX-related side effects in the RCTs included in this analysis were diverse; therefore, we reported these outcomes using ‘standardized units’.^[10]^ Bradycardia and hypotension were reported in 5 trials;^[22–25,27]^ and were reported as absent in 1 trial.^[22]^ Combining DEX with LAs increased the odds of hypotension by an OR [95% CI] of 3.89 [1.35, 11.18], (*P* =.01, I^2^ = 0); however, there was no statistically significant difference in the incidence of bradycardia, with an OR [95% CI] of 3.75 [0.98, 14.31], (*P* =.05, I^2^ = 0), as shown in Figure 6.

Postoperative sedation was reported using various scales, including the Observer's Assessment of Alertness/Sedation (OAA/S) scale^[22,24]^ and Richmond Agitation Sedation Score (RASS).^[23]^ OAA/S scores were significantly higher in the DEX group when compared with the Control group;^[24]^ however, RASS were comparable in the study^[23]^ and detailed data about the sedation scores were not present in another trial.^[22]^

Hypoxemia was defined as oxygen saturation <90%^[22,27]^ or was not defined. None of the patients in the reviewed trials experienced hypoxemic events.

The incidence of PONV was reported in 5 trials.^[22,23,25–27]^ Data revealed no statistically significant difference in the incidence of PONV between the 2 groups, with an OR of PONV incidence [95% CI] of 0.63 [0.32, 1.23], (*P* = .18, I^2^ = 0), as shown in Figure 6.

Finally, complications related to the paravertebral technique were observed in some studies, with pneumothorax^[22,26]^ and vascular puncture^[21]^ occurring in 1 and 1 patient, respectively, during the procedure.

## 4 Discussion

Our systematic review and meta-analysis showed that combining DEX with LAs for PVB significantly improved postoperative pain scores while at rest and dynamic, extended the duration of analgesia, and reduced cumulative postoperative analgesic consumption when compared with LAs alone. These results were similar to the meta-analysis assessing DEX as a LA adjuvant for BPB^[10,12]^ and neuraxial block.^[13]^ Furthermore, the adjuvant DEX did not cause any increased risk of bradycardia or PONV, but led to an increased risk of hypotension. However, the present results are similarly characterized by high heterogeneity. We conducted further subgroup and sensitivity analyses to find the origin of heterogeneity but unfortunately, we failed to identify the source; therefore, our results should be interpreted with caution. Nevertheless, these results provide a firm basis for future, more comprehensive assessment of the use of DEX in combination with LAs in PVB.

The amelioration of clinical outcomes shown in the DEX group may be caused by a peripheral mechanism of action or central effects as the absorption and systemic redistribution of perineurally administered DEX occurs. Fritsch et al^[29]^ measured plasma levels of DEX after perineural administration of 150 μg of DEX with ropivacaine in an interscalene nerve block and concluded that the block-prolonging effects of dexmedetomidine are not systemic in origin. Two volunteer studies^[30,31]^ and 1 animal trial^[32]^ have shown that perineural co-administration of dexmedetomidine and LAs leads to a significantly prolonged nerve block that is attributed to a peripheral mechanism, not systemic effects. The peripheral analgesic mechanism of DEX may be associated with a reduction in the release of norepinephrine and independent inhibition of nerve fiber action potentials *via* the alpha-2 receptor.^[23]^

This meta-analysis has positive safety implications. DEX emerges as a potential adjuvant with a better effect in combination with LAs for adult^[10,13,33]^ and pediatric^[34]^ treatment, including for peripheral nerve and neuraxial blocks. It remains questionable as to whether the magnitude of the difference in the duration of the nerve block between the 2 modes of administration is large enough to warrant off-label perineural use of DEX. Indeed, this applies to all adjuvants because the FDA and European Medicines Agency do not approve of any for perineural use.^[30]^

Pooled analyses showed that DEX increased the incidence of hypotension. This may result from the inhibition of DEX on sympathetic outflow and release of norepinephrine via alpha-2 subtype receptors;^[35]^ however, the reported hypotension was transient and could be reversed by ephedrine. Postoperative sedation was reported in 3 trials, but excessive postoperative sedation was not reported in this analysis. Other adverse effects were comparable in the 2 groups.

Our literature review included all relevant databases and was limited to randomized trials; however, there are several limitations in our study. First, the clinical data originated from different surgical procedures, analgesic drugs, and the level of PVB. In addition, the definition and assessment of some outcomes were inconsistent, which may be the main reason for the observed heterogeneity. Second, the standards of research ethics committees (RECs) were different between studies.^[36]^ DEX was only approved for intravenous delivery by the FDA; therefore all trials were performed in the developing countries, China,^[26,27]^ India,^[21,23,24]^ and Egypt.^[22,25]^ This may be an additional source of publication bias. Third, we excluded conference abstracts and unpublished or in progress trials and only included trials published in the English language. This may impact the clinical heterogeneity of the study. In addition to efficacy, adverse events and hemodynamic safety should be considered when deciding whether to administer dexmedetomidine perineurally or systemically. Further research should focus on the long-term safety and mechanisms of DEX perineural administration.

## 5 Conclusion

In summary, our study concluded that DEX combined with LAs in PVB for appropriate unilateral surgical trunk procedures significantly improved postoperative pain scores while at rest and dynamic, extended the duration of analgesia, and reduced cumulative postoperative analgesic consumption. However, we cannot neglect the heterogeneity of the RCTs included in this analysis. More large-scale prospective studies are needed to further clarify the above conclusions.

## Author Contributions

WK, WLJ, YTJ, MQX, WZ, and CLY conceived and designed the experiments. WK, WLJ, and YTJ performed the experiments. WK, MQX, and WZ analyzed the data. CLY contributed reagents/materials/analysis tools. WK and CLY wrote the paper.
